# Increased CXCL12, a potential CSF biomarker for differential diagnosis of amyotrophic lateral sclerosis

**DOI:** 10.1093/braincomms/fcae271

**Published:** 2024-08-13

**Authors:** Sergio Roca-Pereira, Raúl Domínguez, Isabel Moreno León, María J Colomina, Antonio Martínez Yélamos, Sergio Martínez Yélamos, Mónica Povedano, Pol Andrés-Benito

**Affiliations:** Group of Neurological diseases and Neurogenetics—Bellvitge Biomedical Research Institute (IDIBELL), L’Hospitalet de Llobregat, Barcelona 08907, Spain; Network Centre of Biomedical Research of Neurodegenerative Diseases (CIBERNED), Institute of Health Carlos III, L’Hospitalet de Llobregat, Barcelona 08907, Spain; Group of Neurological diseases and Neurogenetics—Bellvitge Biomedical Research Institute (IDIBELL), L’Hospitalet de Llobregat, Barcelona 08907, Spain; Network Centre of Biomedical Research of Neurodegenerative Diseases (CIBERNED), Institute of Health Carlos III, L’Hospitalet de Llobregat, Barcelona 08907, Spain; Functional Unit of Amyotrophic Lateral Sclerosis (UFELA), Service of Neurology—Bellvitge University Hospital (HUB), L’Hospitalet de Llobregat, Barcelona 08907, Spain; Group of Neurological diseases and Neurogenetics—Bellvitge Biomedical Research Institute (IDIBELL), L’Hospitalet de Llobregat, Barcelona 08907, Spain; Department of Neurology, Multiple Sclerosis Unit, Bellvitge University Hospital, L'Hospitalet de Llobregat, Barcelona 08907, Spain; Anesthesia and Critical Care Department, Bellvitge University Hospital—University of Barcelona (UB), L'Hospitalet de Llobregat, Barcelona 08907, Spain; Group of Neurological diseases and Neurogenetics—Bellvitge Biomedical Research Institute (IDIBELL), L’Hospitalet de Llobregat, Barcelona 08907, Spain; Department of Neurology, Multiple Sclerosis Unit, Bellvitge University Hospital, L'Hospitalet de Llobregat, Barcelona 08907, Spain; Departament de Ciències Clíniques, Facultat de Medicina i Ciències de la Salut, Universitat de Barcelona (UB), L'Hospitalet de Llobregat, Barcelona 08907, Spain; Group of Neurological diseases and Neurogenetics—Bellvitge Biomedical Research Institute (IDIBELL), L’Hospitalet de Llobregat, Barcelona 08907, Spain; Department of Neurology, Multiple Sclerosis Unit, Bellvitge University Hospital, L'Hospitalet de Llobregat, Barcelona 08907, Spain; Departament de Ciències Clíniques, Facultat de Medicina i Ciències de la Salut, Universitat de Barcelona (UB), L'Hospitalet de Llobregat, Barcelona 08907, Spain; Group of Neurological diseases and Neurogenetics—Bellvitge Biomedical Research Institute (IDIBELL), L’Hospitalet de Llobregat, Barcelona 08907, Spain; Network Centre of Biomedical Research of Neurodegenerative Diseases (CIBERNED), Institute of Health Carlos III, L’Hospitalet de Llobregat, Barcelona 08907, Spain; Functional Unit of Amyotrophic Lateral Sclerosis (UFELA), Service of Neurology—Bellvitge University Hospital (HUB), L’Hospitalet de Llobregat, Barcelona 08907, Spain; Group of Neurological diseases and Neurogenetics—Bellvitge Biomedical Research Institute (IDIBELL), L’Hospitalet de Llobregat, Barcelona 08907, Spain; Network Centre of Biomedical Research of Neurodegenerative Diseases (CIBERNED), Institute of Health Carlos III, L’Hospitalet de Llobregat, Barcelona 08907, Spain

**Keywords:** ALS, diagnostic tool, CXCL12, CSF

## Abstract

Amyotrophic lateral sclerosis is a debilitating and lethal neurodegenerative disorder marked by the gradual deterioration of motor neurons. Diagnosing amyotrophic lateral sclerosis is challenging due to the lack of reliable diagnostic tools, with clinical assessment being the primary criterion. Recently, increased levels of neurofilament light chain in CSF have been considered a useful biomarker in disease, correlating with disease progression but not specific for diagnosis. This study utilized enzyme-linked immunosorbent assay to measure CSF C-X-C motif chemokine ligand 12 levels in healthy controls, amyotrophic lateral sclerosis patients and patients with amyotrophic lateral sclerosis–mimic disorders, assessing its potential as a diagnostic biomarker and comparing it with neurofilament light chain levels. Our results confirmed previous findings, showing increased C-X-C motif chemokine ligand 12 levels in amyotrophic lateral sclerosis patients compared to healthy control (797.07 ± 31.84 pg/mL versus 316.15 ± 16.6 pg/mL; *P* = 0.000) and increased CSF neurofilament light chain levels in amyotrophic lateral sclerosis (4565.63 ± 263.77 pg/mL) compared to healthy control (847.86 ± 214.37 pg/mL; *P* = 0.000). Increased C-X-C motif chemokine ligand levels were specific to amyotrophic lateral sclerosis, not seen in amyotrophic lateral sclerosis–mimic conditions like myelopathies (252.20 ± 23.16 pg/mL; *P* = 0.000), inflammatory polyneuropathies (270.24 ± 32.23 pg/mL; *P* = 0.000) and other mimic diseases (228.91 ± 29.20 pg/mL; *P* = 0.000). In contrast, CSF neurofilament light chain levels in amyotrophic lateral sclerosis overlapped with those in myelopathies (2900.11 ± 872.20 pg/mL; *P* = 0.821) and other mimic diseases (3169.75 ± 1096.65 pg/mL; *P* = 0.63), but not with inflammatory polyneuropathies (1156.4 ± 356.6 pg/mL; *P* = 0.000). Receiver operating characteristic curve analysis indicated significant differences between the area under the curve values of C-X-C motif chemokine ligand and neurofilament light chain in their diagnostic capacities. C-X-C motif chemokine ligand could differentiate between amyotrophic lateral sclerosis and myelopathies (area under the curve 0.99 ± 0.005), inflammatory polyneuropathies (area under the curve 0.962 ± 0.027) and other mimic diseases (area under the curve 1.00 ± 0.00), whereas neurofilament light chain was only effective in inflammatory polyneuropathies cases (area under the curve 0.92 ± 0.048), not in myelopathies (area under the curve 0.71 ± 0.09) or other mimic diseases (area under the curve 0.69 ± 0.14). We also evaluated C-X-C motif chemokine ligand levels in plasma [amyotrophic lateral sclerosis (2022 ± 81.8 pg/mL) versus healthy control (1739.43 ± 77.3 pg/mL; *P* = 0.015)] but found CSF determination (area under the curve 0.97 ± 0.012) to be more accurate than plasma determination (area under the curve 0.65 ± 0.063). In plasma, single molecule array (SIMOA) neurofilament light chain determination [amyotrophic lateral sclerosis (86.00 ± 12.23 pg/mL) versus healthy control (12.69 ± 1.15 pg/mL); *P* = 0.000] was more accurate than plasma C-X-C motif chemokine ligand 12 (area under the curve 0.98 ± 0.01405). These findings suggest that CSF C-X-C motif chemokine ligand 12 levels can enhance diagnostic specificity in distinguishing amyotrophic lateral sclerosis from amyotrophic lateral sclerosis–mimic disorders, compared to neurofilament light chain. Larger studies are needed to validate these results, but C-X-C motif chemokine ligand 12 determination shows promising diagnostic potential.

## Teaser

Roca-Pereira *et al*. found C-X-C motif chemokine ligand 12 (CXCL12) more specific for amyotrophic lateral sclerosis compared to neurofilament light chain (NF-L), the current gold standard biomarker. While NF-L is altered in many pathologies, CXCL12’s specificity underscores its relevance in amyotrophic lateral sclerosis pathophysiology and its potential utility in differential diagnosis of the disease.

## Graphical Abstract

**Graphical Abstract:**
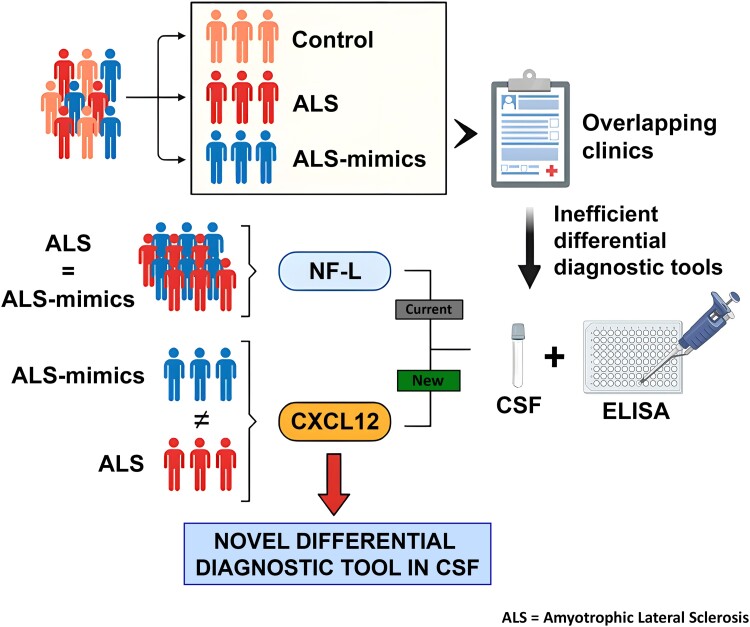

## Introduction

Amyotrophic lateral sclerosis is a debilitating and lethal neurodegenerative disorder marked by the gradual deterioration of both upper (UMN) and lower motor neurons (LMN).^1^ Despite intensive research, current management of amyotrophic lateral sclerosis remains suboptimal from diagnosis to prognosis. When a patient presents with a progressive upper and/or lower motor syndrome, clinicians must pay particular attention to any atypical features in the history and/or clinical examination suggesting an alternate diagnosis, as up to 10% of patients initially diagnosed with amyotrophic lateral sclerosis have a mimic of amyotrophic lateral sclerosis.^2^

Diagnosing ALS is a complex process as there is no single test or procedure that definitively confirms the presence of the disease. It is a diagnosis of exclusion, requiring the elimination of other conditions that may mimic ALS symptoms, before a conclusive diagnosis can be established.^3,4^ Confusion conditions that most commonly mimic amyotrophic lateral sclerosis include multifocal motor neuropathy with conduction block, axonal motor predominant chronic inflammatory demyelinating polyneuropathy, spinobulbar muscular atrophy and inclusion body myositis. Simultaneous cervical nerve root and spinal cord compression by disc herniations, tumours or malformations may cause combined LMN signs in the arms and UMN in the legs and be misdiagnosed as classical amyotrophic lateral sclerosis. Upper motor neuron–dominant amyotrophic lateral sclerosis or primary lateral sclerosis may be confused with hereditary spastic paraplegias or primary progressive multiple sclerosis. Additional, but rare, differential diagnoses include hyperparathyroidism and hexosaminidase A/B deficiency. Since some of these conditions are treatable, it is important to rule out these possibilities.^5^

Insights into the pathophysiology of amyotrophic lateral sclerosis; identification of disease biomarkers and modifiable risks, along with new predictive models, scales and scoring systems; and a clinical trial pipeline of mechanism-based therapies are changing the prognostic landscape, although most recent advances have yet to translate into patient benefit. Nowadays, only increased levels of neurofilaments (NFs) in the CSF are considered the most useful biomarker in amyotrophic lateral sclerosis.^6-9^ However, increased levels of NF-L in the CSF are not specific to the disease, being increased in most of the neurological conditions.^10-17^ Given the lack of specificity in NF-L, our group has previously sought to identify novel molecules that could enhance the diagnosis of amyotrophic lateral sclerosis. Our recent work suggests that assessing C-X-C motif chemokine ligand 12 (CXCL12) levels in CSF could serve as a valuable complementary diagnostic biomarker for amyotrophic lateral sclerosis, but not as a prognostic biomarker.^18^ In the context of other neurological conditions, our findings revealed a significant increase in CXCL12 levels in the CSF of MS patients but remained unaltered in other neurodegenerative conditions as assessed in our comparative study.^18^ This data in MS indicated that is not a specific marker for amyotrophic lateral sclerosis. However, the interpretation of biochemical data is always contextualized within the clinical manifestations of individual patients. Therefore, these findings suggest that CXCL12 may serve as a valuable complementary diagnostic biomarker in amyotrophic lateral sclerosis. In the present study, we are aimed to unveil CXCL12’s potential in the differential diagnosis of conditions mimicking amyotrophic lateral sclerosis symptoms and compare its capacity with the current gold standard, the CSF NF-L levels.

## Materials and methods

### CSF and plasma collection

Amyotrophic lateral sclerosis was diagnosed according to updated El Escorial criteria.^3,4^ The Revised Amyotrophic Lateral Sclerosis Functional Rating Scale (2015) was used in every case through the clinical course of the disease. CSF was obtained at the first visit of diagnosis, with an average time collection sample of 10 months after the onset of symptoms. CSF was prospectively collected from patients undergoing lumbar puncture (LP) due to clinical suspicion of MN disease at the Neurology Service of the Bellvitge University Hospital, including amyotrophic lateral sclerosis–mimic diseases cases. In these patients, 1.5 ± 0.5 mL of CSF was collected in polypropylene tubes and centrifuged at 3000 rpm for 15 min at room temperature. The supernatant was collected and aliquoted in volumes of 220 μL and stored at −80°C until use. Amyotrophic lateral sclerosis cases did not carry *C9ORF72* expansions, *SOD1* and *TARDBP* mutations. CSF from healthy controls (HCs) was obtained from patients at the need for knee surgical procedures under spinal anaesthesia. The studied cohort includes CSF from 48 sporadic amyotrophic lateral sclerosis cases and 68 healthy donors. As amyotrophic lateral sclerosis–mimic disorders, we included 17 myelopathy cases, 26 inflammatory polyneuropathy (IP) cases and other amyotrophic lateral sclerosis–mimic diseases (OMD) that includes four sensitive-motor syndrome cases and three paraparesis cases. Cases and controls had not suffered from infection or inflammatory diseases at the time of sampling. In addition, plasma paired samples of 36 HC and 43 amyotrophic lateral sclerosis cases previously analysed for CSF CXCL12 quantification^18^ were collected using the same processing protocol. Cases are summarized in Table 1. Samples were obtained according to the Declaration of Helsinki and following informed consent and approval by the Clinical Research Ethics Committee of the Hospital (Ref. PR010/22).

**Table 1:** Summary of cases amyotrophic lateral sclerosis and amyotrophic lateral sclerosis–mimic disorders used for CXCL12 determination

Group	*N*	Age (years)	Sex (F/M)	ALS Onset
CSF				
HC	68	68 ± 10.8	(39/29)	
ALS	48	65 ± 12.09	(23/25)	31 Spinal
				16 Bulbar
				1 Resp.
IP	25	50 ± 20.1	(8/17)	
Myelopathy	17	51 ± 17.6	(11/6)	
OMD	7	42 ± 13.8	(3/4)	
Plasma				
HC	36	68.1 ± 10	(17/19)	
ALS	41	61.8 ± 11.5	(26/15)	20 Spinal
				21 Bulbar

ALS, amyotrophic lateral sclerosis; CSF, cerebrospinal fluid; F, female; IP, inflammatory polyneuropathy; HCs, healthy controls; M, male; Myel, myelopathy; OMDs, other mimic diseases.

### Biofluid analysis

CXCL12 was quantified using the Human CXCL12/SDF-1α Quantikine ELISA Kit from R&D Systems according to the manufacturer’s instructions (R&D Systems, Inc., MN, USA). Neurofilament light chain levels were quantified using the NF-Light ELISA for CSF samples (10-7001 CE) from UmanDiagnostics (Umea, Sweden), approved for diagnostic purposes, and single molecule array (SIMOA) NF-Light Advantage Kit for plasma samples determination from Quanterix (Billerica, MA, USA), according to the manufacturer’s instructions. Test performers were blinded to clinical information.

### Statistical analysis

The statistical analysis and graphic design were performed using the GraphPad Prism software version 9.5.0 (La Jolla, CA, USA). The normality of distribution was analysed with the Shapiro–Wilk test. The statistical analysis between sample groups was carried out using a Kruskal–Wallis test followed by Dunn’s multiple comparisons test. Receiver operating characteristic (ROC) curves and derived area under the curve (AUC) were calculated. Best cut-off value, sensitivity and specificity were estimated based on the Youden index; and ROC curve comparisons were determined with DeLong’s method. Biomarker combination assessment was performed using CombiROC free software. The unpaired Student’s *t*-test or Mann-Whitney test was used to compare plasma samples data. Pearson’s or Spearman's correlation were used to assess associations between biomarker levels. Outliers were detected using the GraphPad software QuickCalcs (*P* < 0.05). The data were expressed as mean ± SEM; significance levels were set at *P < 0.05 and ***P < 0.0001.

## Results

### The potential of CXCL12 as a clinical tool for the differential diagnosis of amyotrophic lateral sclerosis and amyotrophic lateral sclerosis–mimics using CSF samples at the time of diagnosis

**Figure 1:**
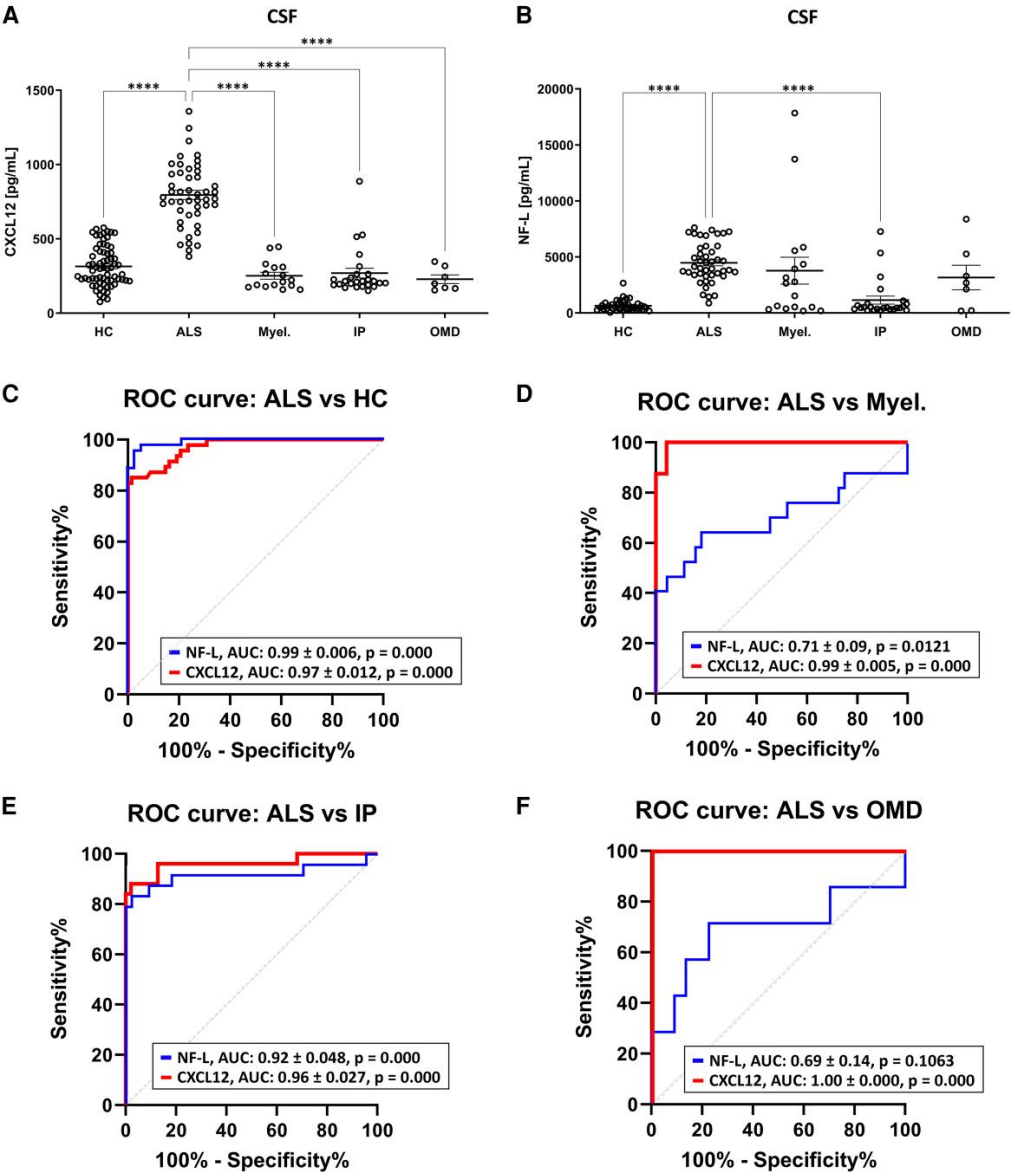
**Differential diagnostic capacity of CSF CXCL12 in ALS.** (**A**) CSF CXCL12 levels in HC (*n* = 68), amyotrophic lateral sclerosis (*n* = 48) and amyotrophic lateral sclerosis–mimic cases (IP, *n* = 25; Myel, *n* = 17; OMD, *n* = 7). Differences between groups were assessed with Kruskal–Wallis test. (**B**) CSF NF-L levels in HC (*n* = 39), amyotrophic lateral sclerosis (*n* = 48) and amyotrophic lateral sclerosis–mimic cases (IP, *n* = 25; Myel, *n* = 17; OMD, *n* = 7). Mean ± SEM is represented in the graphs. (**C**) ROC curve of CXCL12 and NF-L in amyotrophic lateral sclerosis as a diagnostic biomarker when comparing with HC. (**D**) ROC curves of CSF CXCL12 and NF-L levels as a differential diagnostic biomarker when comparing amyotrophic lateral sclerosis (*n* = 48) with myelopathies (*n* = 17) as amyotrophic lateral sclerosis–mimic diseases. (**E**) ROC curves of CSF CXCL12 and NF-L levels as a differential diagnostic biomarker when comparing ALS (*n* = 48) with IP (*n* = 25) as ALS-mimic diseases. (**F**) ROC curves of CSF CXCL12 and NF-L levels as a differential diagnostic biomarker when comparing ALS (*n* = 48) with OMD (*n* = 7) as amyotrophic lateral sclerosis–mimic diseases. Significance was set at *P < 0.05 and ****P < 0.0001. AUC, area under the curve; CSF, cerebrospinal fluid; IP, inflammatory polyneuropathy; HCs, healthy controls; Myel, myelopathy; OMDs, other mimic diseases; ROC, receiver operating characteristic.

CSF CXCL12 levels were quantified in our cohort of cases showing significant differences among groups (KW = 94.25, *P* = 0.000). Significantly higher CXCL12 protein levels were detected in amyotrophic lateral sclerosis (797.07 ± 31.84 pg/mL) when compared to HC (316.15 ± 16.6 pg/mL; *P* = 0.000) and amyotrophic lateral sclerosis–mimic diseases, such as myelopathy (252.20 ± 23.16 pg/mL; *P* = 0.000), IP (270.24 ± 32.23 pg/mL; *P* = 0.000) and OMD (228.91 ± 29.20 pg/mL; *P* = 0.000) cases (Fig. 1A). To be able to compare with the main stablished biomarker, CSF NF-L levels were quantified in our cohort of cases showing significant differences among groups (KW = 63.51, *P* = 0.000). Significantly higher NF-L protein levels were detected in amyotrophic lateral sclerosis (4565.63 ± 263.77 pg/mL) when compared to HC (847.86 ± 214.37 pg/mL; *P* = 0.000) and also when compared to IP (1156.4 ± 356.6 pg/mL; *P* = 0.000) but not when compared to amyotrophic lateral sclerosis–mimic diseases, such as myelopathy (2900.11 ± 872.20 pg/mL; *P* = 0.821) and the OMD (3169.75 ± 1096.65 pg/mL; *P* = 0.63) cases (Fig. 1B).

To calculate the diagnostic accuracy of CSF CXCL12 in discriminating between HC and amyotrophic lateral sclerosis cases, we estimated the corresponding AUC value. Receiver operating characteristic analysis indicated an AUC value of 0.97 ± 0.012 (95% CI 0.95–0.9959, *P* = 0.000) for amyotrophic lateral sclerosis, with the optimal cut-off at 568.3 pg/mL determined by the Youden index (0.83; sensitivity of 85.11%; specificity of 98.53%; Fig. 1C). Similarly, to calculate the diagnostic accuracy of CSF NF-L and compare to CXCL12’s accuracy in discriminating between HC and amyotrophic lateral sclerosis cases, we estimated the corresponding AUC value. Receiver operating characteristic (ROC) curve analysis indicated an AUC value for NF-L of 0.99 ± 0.006 (95% CI 0.98–1.000, *P* = 0.000) for amyotrophic lateral sclerosis, with the optimal cut-off at 1513 pg/mL determined by the Youden index (0.92; sensitivity of 95.45%; specificity of 97.37%; Fig. 1C). The comparison between the AUC values of both biomarkers using DeLong’s method determined that there are not significant differences between CXCL12 and NF-L (ΔArea 0.02 ± 0.0206, *z* = −9725, *P* = 0.33) in diagnosis accuracy, showing similar capabilities to differentiate between HC and amyotrophic lateral sclerosis cases.

However, to test the potential capacity of CSF CXCL12 levels to differentiate between amyotrophic lateral sclerosis and amyotrophic lateral sclerosis–mimic cases, we also estimated the corresponding AUC values for each biomarker. For CXCL12, ROC analysis indicated an AUC value of 0.99 ± 0.005 (95% CI 0.98–1.00, *P* = 0.000) for myelopathies, with the optimal cut-off at 451.1 pg/mL determined by the Youden index (0.95; sensitivity of 100%; specificity of 95.7%; Fig. 1D). Another ROC analysis indicated an AUC value of 0.962 ± 0.027 (95% CI 0.90–1.00, *P* = 0.000) for IP, with the optimal cut-off at 409.1 pg/mL determined by the Youden index (0.86; sensitivity of 88%; specificity of 97.87%; Fig. 1E). Finally, ROC analysis indicated an AUC value of 1.00 ± 0.00 (95% CI 1.00–1.00, *P* = 0.000) for OMD, with the optimal cut-off at 364.4 pg/mL determined by the Youden index (1.00; sensitivity of 100%; specificity of 100%; Fig. 1F).

To compare differential diagnosis ability between CSF CXCL12 levels and CSF NF-L levels, we also calculated the AUC values for NF-L in the same cohort of amyotrophic lateral sclerosis patients and amyotrophic lateral sclerosis–mimic disorders. Receiver operating characteristic analysis indicated an AUC value of 0.71 ± 0.09 (95% CI 0.53–0.886, *P* = 0.0121) for myelopathies, with the optimal cut-off at 3219 pg/mL determined by the Youden index (0.46; sensitivity of 64.71%; specificity of 81.82%; Fig. 1D). Another ROC analysis indicated an AUC value of 0.92 ± 0.048 (95% CI 0.82–1.00, *P* = 0.000) for IP, with the optimal cut-off at 1273 pg/mL determined by the Youden index (0.81; sensitivity of 88%; specificity of 97.87%; Fig. 1E). Finally, ROC analysis indicated an AUC value of 0.69 ± 0.14 (95% CI 0.4188–0.9643, *P* = 0.1063) for OMD, with the optimal cut-off at 3338 pg/mL determined by the Youden index (0.48; sensitivity of 71.43%; specificity of 77.3%; Fig. 1F).

Finally, AUC values of both analytes were compared for each disease. Significant differences were observed between CXCL12 and NF-L accuracy in the differential diagnosis between amyotrophic lateral sclerosis and amyotrophic lateral sclerosis–mimic disease, with CXCL12 being significantly more accurate in differentiating myelopathies (ΔArea 0.2861 ± 0.0684, *z* = 4.1841, *P* = 0.000; Fig. 1D) and OMD (ΔArea 0.3084 ± 0.096, *z* = 3.211, *P* = 0.0013; Fig. 1F). In the case of IP, although the AUC value was higher for CXCL12, no significant differences were reported when comparing the accuracy of NF-L or CXCL12 CSF levels (ΔArea 0.0431 ± 0.038, *z* = 1.1343, *P* = 0.257; Fig. 1E).

### Levels of CXCL12 in plasma samples do not enhance the diagnostic capability of CSF CXCL12 levels or the accuracy of plasma neurofilament light chain

Based on CSF results, we decided to evaluate the diagnostic ability of CXCL12 levels in plasma, a more accessible biofluid, and compare to NF-L’s accuracy to differentiate between HC and amyotrophic lateral sclerosis patients. CXCL12 and NF-L determination was not able to be performed on plasma samples of amyotrophic lateral sclerosis–mimic cases due to the lack of biobanked samples from these patients, as the collection and biobanking of plasma samples are outside the hospital’s protocol for the differential diagnosis, which only includes CSF obtention.

**Figure 2:**
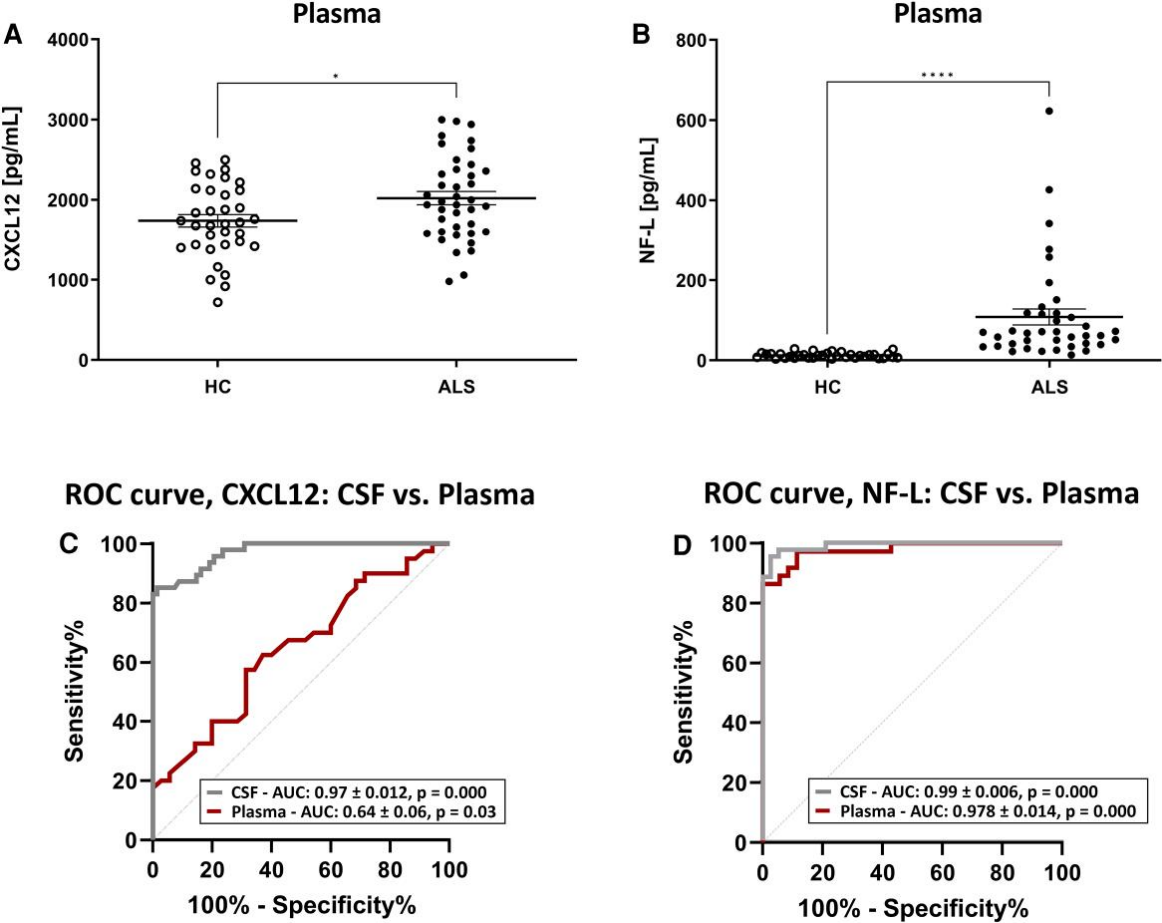
**Diagnostic capacity of plasma CXCL12 levels in ALS.** Plasma levels of (**A**) CXCL12 and (**B**) NF-L in HC (*n* = 36) and amyotrophic lateral sclerosis cases (*n* = 41). Differences between groups were assessed with Student’s *t*-test and Mann-Whitney test, respectively. (**C**) ROC curves analysis of CXCL12 in CSF and plasma. (**D**) ROC curves analysis of NF-L in CSF and plasma. AUC, area under the curve; CSF, cerebrospinal fluid; HCs, healthy controls; ROC, receiver operating characteristic.

*T*-test indicated a significant increase when comparing plasma levels of CXCL12 in amyotrophic lateral sclerosis cases (2022 ± 81.8 pg/mL) to HC cases (1739.43 ± 77.3 pg/mL; *P* = 0.015; Fig. 2A). For NF-L levels in plasma samples, Mann–Whitney test also indicated significant differences (MW = 28, *P* = 0.000), showing a significant increase when comparing plasma levels in amyotrophic lateral sclerosis cases (86.00 ± 12.23 pg/mL) to HC cases (12.69 ± 1.15 pg/mL; *P* = 0.000; Fig. 2B).

To calculate the diagnostic accuracy of plasma CXCL12 in discriminating between HC and amyotrophic lateral sclerosis cases, we estimated the corresponding AUC value. Receiver operating characteristic analysis indicated an AUC value of 0.65 ± 0.063 (95% CI 0.52–0.77, *P* = 0.03) for plasma CXCL12 when comparing ALS and HC cases, with the optimal cut-off at 1910 pg/mL determined by the Youden index (0.26; sensitivity of 57.5%; specificity of 68.6%; Fig. 2C). For plasma NF-L levels, ROC analysis indicated an AUC value of 0.98 ± 0.01405 (95% CI 0.95–1.00, *P* = 0.000) when comparing amyotrophic lateral sclerosis and HC cases, with the optimal cut-off at 29.03 pg/mL determined by the Youden index (0.865; sensitivity of 86.5%; specificity of 100%; Fig. 2D). The comparison between CXCL12 and NF-L AUC values in plasma determined that there were significant differences between both molecules’ accuracy (ΔArea 0.325 ± 0.0657, *z* = −4.95, *P* = 0.000), being significantly more accurate the plasma NF-L determination than CXCL12 determination.

The comparison between CXCL12 biofluids’ AUC values determined that there were significant differences between CSF and plasma accuracy (ΔArea 0.325 ± 0.0657, *z* = −4.95, *P* = 0.000), being the CSF determination significantly more accurate than the plasma determination. In contrast, NF-L biofluids’ AUC values determined that there were not significant differences between CSF and plasma accuracy (ΔArea 0.0138 ± 0.0198, *z* = 0.6982, *P* = 0.485; Fig. 2D). Finally, based on these results, we decided to add the accuracy of both biomarkers in a two-biomarker panel to increase the single potential of each one using CombiRoc software for ALS diagnosis using CSF data, but due to its lack of AUC differences and differences in unit orders, no significant improvement was achieved (combined biomarkers, 84% sensitivity and 86% specificity).

### Levels of CXCL12 biofluids do not correlate with neurofilament light chain levels or Revised Amyotrophic Lateral Sclerosis Functional Rating Scale scores

Finally, the relationship between CSF and plasma CXCL12 levels was studied using Pearson correlation regression, but no significant relationship was determined between the two biofluids (Pearson *r* = 0.1413, *P* = 0.2234). In amyotrophic lateral sclerosis cases, the relationship between CXCL12 and NF-L levels in CSF and plasma was also assessed, but no significant correlations were observed (Pearson *r* = 0.08784, *P* = 0.5754; and Spearman *r* = −0.1093, *P* = 0.5195, respectively). Furthermore, both biomarkers’ levels were correlated with Revised Amyotrophic Lateral Sclerosis Functional Rating Scale (ALS-FRS-R) clinical scores. For CXCL12, no significant associations were reported between CXCL12 levels and ALS-FRS-R clinical either in CSF (Spearman *r* = 0.01107, *P* = 0.9425) or plasma (Spearman *r* = 0.064, *P* = 0.7317). In contrast, as expected, CSF and plasma NF-L levels correlated with ALS-FRS-R clinical scores (Spearman *r* = 0.4873, *P* = 0.0009; and Spearman r = 0.72, *P* = 0.000, respectively).

## Discussion

Amyotrophic lateral sclerosis diagnosis is challenging. Clinicians use the El Escorial, Awaji, or Gold Coast criteria as primary diagnostic tools. Following these, clinicians conduct physical and neurological exams and immunological profiling and review medical history to rule out treatable conditions mimicking amyotrophic lateral sclerosis. However, no single test definitively establishes amyotrophic lateral sclerosis diagnosis. It often involves a ‘rule-out’ procedure, confirmed only after eliminating other conditions through specific tests, reducing confirmation certainty. Thus, it is extremely important not to miss little cues or use novel tools which can suggest an alternative diagnosis and in many cases a lease of life in terms of a treatment option.

Considering this, the development of biomarkers for understanding amyotrophic lateral sclerosis and establishing progression and therapeutic target engagement are a crucial objective for disease management. In our study, we contributed to this goal by quantifying CSF CXCL12 levels of amyotrophic lateral sclerosis patients and comparing them with those of HCs and patients with amyotrophic lateral sclerosis–mimicking diseases. Our results, building on previous findings in amyotrophic lateral sclerosis compared to other neurodegenerative conditions,^17^ highlight that CSF CXCL12 levels serve as an excellent tool for distinguishing between amyotrophic lateral sclerosis and diseases with overlapping clinical presentations, such as paraparesis, sensitive-motor syndrome, myelopathy and inflammatory polyneuropathy, improving or equalizing NF-L determinations that are more unspecific, being commonly altered in this type of diseases,^14,15^ similarly as occurs in the use of other biomarkers such as chitinases or GPNMB protein, among others.^19-21^ Thus, our results are not intended to displace the use of NF-L in CSF, but rather to complement it, and improve the specificity for neurodegenerative diseases or clinically mimicking amyotrophic lateral sclerosis.

Furthermore, we validated the assessment of plasma CXCL12 levels in amyotrophic lateral sclerosis, providing a cost-effective and non-invasive alternative suitable for broader use, particularly in serial collections. However, it is essential to note that the accuracy of this biomarker in plasma is somewhat lower than in CSF, and its accuracy is lower than plasma NF-L levels. The lack of paired plasma samples of amyotrophic lateral sclerosis–mimic disorders does not allow us to define the capability of CXCL12 in the differential diagnosis of amyotrophic lateral sclerosis, only amyotrophic lateral sclerosis diagnosis, and neither compare it with NF-L. However, differential diagnosis procedures in clinics for this type of amyotrophic lateral sclerosis–mimic disorders require the LP procedure to assess multiple possible diagnoses, so the use of CSF is justified. Therefore, we emphasize that this analysis should be conducted in the first biofluid following a LP procedure—a safe method for ALS patients, with a comparable adverse event (AE) rate than observed in patients without ALS.^22^

Hence, the measurement of CSF CXCL12 levels could be regarded as a valuable parameter in the diagnostic process of ALS. Its inclusion in the ALS diagnostic routine should be contemplated, as it has the potential to enhance specificity, complementing the determination of NF-L levels, which demonstrates high sensitivity but lacks specificity. Moreover, the data obtained in this cohort indicated that NF-L and CXCL12 do not correlate with each other in either biofluid. No association has been demonstrated between CSF and plasma CXCL12 levels and ALS-FRS-R scores, unlike the correlation seen with NF-L levels in CSF and plasma, as previously has been observed.^23,24^ In consequence, more studies involving larger cohorts of patients and exploration of other mimic conditions are necessary to validate and strengthen our findings. Nonetheless, the promising applications of CXCL12 determination suggest its potential significance in diagnostic practices.

Besides, these results reinforce the specific role of CXCL12 in ALS pathophysiology. Despite presenting similar phenotypes or sharing mechanisms implicated in neurodegenerative processes or MN degeneration,^18^ or clinical overlap, our data demonstrate that the molecular signature is not uniform in each case, indicating specificity of CXCL12 pathway alteration in ALS context. Thus, beyond being a new clinical tool candidate, the study of the role of CXCL12 could unveil novel physiopathological pathways and potential therapeutic targets or surrogate markers for treatment response.

## Data Availability

Data related to the findings presented in this paper are available from the corresponding author upon reasonable request.
